# Vocal greeting during mother-infant reunions in a nocturnal primate, the gray mouse lemur (*Microcebus murinus*)

**DOI:** 10.1038/s41598-017-10417-8

**Published:** 2017-09-04

**Authors:** Marina Scheumann, Sabrina Linn, Elke Zimmermann

**Affiliations:** Institute of Zoology, University of Veterinary Medicine Hannover, Bünteweg 17, Hannover, D-30559 Germany

## Abstract

In human societies, ritualized greeting behavior includes gestural and vocal displays to signal the social acceptance of an encountering person. These displays are universal across cultures suggesting a pre-human origin. Vocal greeting displays are only reported for monkeys and apes with complex social systems, but none of these studies confirmed that greeting signals fulfill all criteria characterizing human greeting behavior. In this study, we analyzed for the first time whether vocal exchanges between mother and infants in a non-human primate fulfill the criteria of human greeting behavior and whether vocal greeting behavior is present in a basal primate with a less complex social system, the gray mouse lemur. By comparing spontaneous leave-takings and reunions, we found that vocal exchanges during mother-infant reunions fulfilled all six criteria characterizing human greeting behavior. Thus, predictable reciprocal vocal exchanges occurred at the start of the reunion (but not during leave-taking), when mother and infant had visual contact to each other. Thus, we argued that mother-infant vocal exchanges governing reunions are essential to establish social bonds and to ritualize the greeting function. Our findings suggest that ritualized vocal greeting has its origins deeply rooted in mammalian phylogeny and derives from vocal exchanges during parent-infant reunions.

## Introduction

Human “greeting” rituals include complex and individual patterns made up of several gestures often combined with linguistic and non-linguistic vocal displays (e.g., “hello” or laughter) to signal the social acceptance of an encountering person^1–5^. According to Duranti^2^ the human “greeting” is defined by six criteria: (1) *context of encounter* – greetings have to occur at an encounter and have to be different from behavioral displays during leave-taking, (2) *shared perceptual field* – the greeting occurs when both interaction partners visually or acoustically (e.g., telephone) recognize each other, (3) *reciprocal exchange* – a greeting is an exchange between both interactors testing the relationship to each other, (4) *predictability* – greeting displays are relatively predictable in form and content, (5) *temporal unit of interactions* – the greeting occurs at the beginning of an interaction and (6) *identification of interlocutor* – the greeting allows an identification of a group membership (e.g., greeting pattern differs depending on the relationships between the two persons e.g., friend or stranger, mother or infant^6, 7^). Human greeting rituals are often multi-modal communicative displays involving acoustic, visual and tactile sensory modalities^2–5^, however, each modality can also be used independently. Thus, for example telephone openings^8^ rely only on the acoustic modality. Such vocal greetings occur across different cultures and languages^2, 3^ and are the first words children acquire (English/Italian children^9^; Danish children^10^) or we learn in a foreign language^2^. This universality suggests a pre-human origin^2, 3^ and leads to the question whether vocal greeting rituals following the same behavioral patterns and functions as in humans can be found in non-human primates^11^, our closest living relatives.

In nonhuman mammals reciprocal greeting displays are reported for the visual and tactile modality including facial expressions, mounting, embracing, and erected penis displays (e.g., baboons^12–14^, spider monkey^15, 16^, hyaena^17, 18^). In contrast, our knowledge on the existence of reciprocal vocal greeting displays is very limited (e.g. chimpanzees^11, 19, 20^, howler monkeys^21^, macaques^22^). None of these studies confirmed that the so-called vocal greeting signals fulfill the criteria of human greeting behavior as suggested by Duranti^2^. Moreover, in chimpanzees vocal reciprocal displays were observed in only 12% of the interactions^11^ concluding that these signals do not fulfill the criteria of *reciprocity*. Thus, it is unclear whether vocal greeting displays are present in non-human mammals.

The aim of this study was to explore whether vocal exchanges between mother and infant gray mouse lemurs at the sleeping site fulfill the criteria of vocal greeting according to Duranti^2^. Mouse lemurs are ideal primate models to investigate the evolutionary roots of vocal greeting behavior: (1) Due to their nocturnal life style the vocal modality was found to play an important role for communication^23^. Thus, vocalizations convey kin-, group- and individual-specific signatures^24, 25^ and are used in a broad variety of contexts^26^. (2) Mouse lemurs show an infant-parking system^27–29^ (i.e. while the mother forages solitarily, she parks her infants in either tree holes or dense vegetation), have a rapid life history^30, 31^ and can be bred successfully in captivity^30^. This allows to model natural situations by analyzing leave-takings and mother-infant reunions at the sleeping site using a standardized captive setting. (3) They are socially disperse living primates (i.e. solitary foragers where females form kin-related sleeping groups during the daytime^30^). This allows investigating whether vocal greeting already occurs in a transition state between solitary ranging and gregariousness or is limited to taxa with highly complex social systems such as chimpanzees. (4) Mouse lemurs are described as basal primates representing an ancestral primate model^28^. Thus, this allows investigating whether vocal greeting already evolved at the basis of the primate stock.

**Figure 1:**
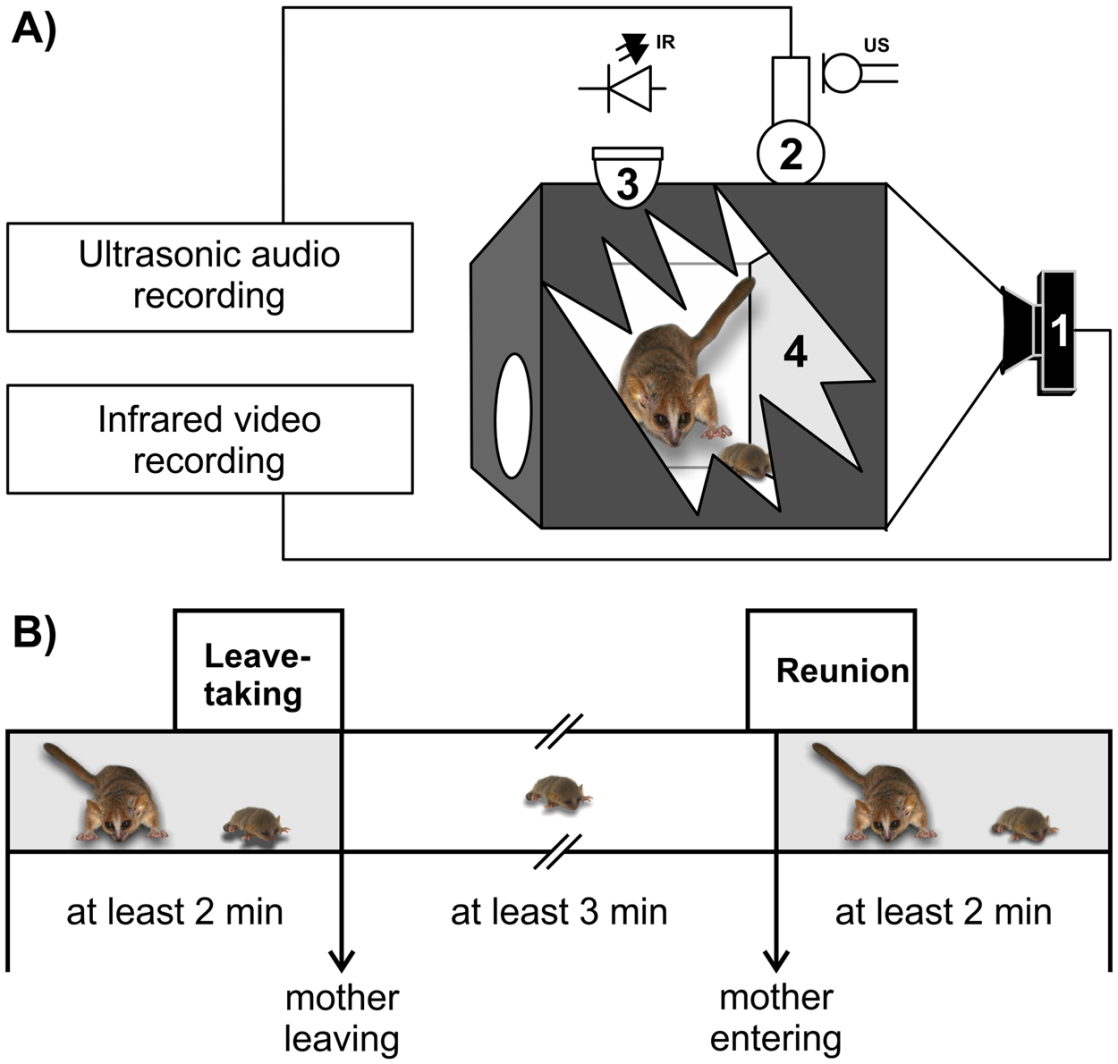
Experimental set-up and definition of Leave-taking and Reunion condition: (**A**) Experimental set-up: 1 = infrared camera connected to video recording device, 2 = ultrasonic microphone connected to audio recording device, 3 = infrared LED to illuminate the sleeping box, 4 = transp﻿arent plastic wall for video recording; (**B**) Time line for the definition of the one-minute Leave-taking (=60 seconds before a mother leaves the nest) and Reunion (=started the last 10 seconds before the mother entered the nest box and finished 50 seconds after entering) observation intervals (OI).

We analyzed vocal exchanges between mothers and their infants during observation intervals (OI) of one minute for two conditions: Leave-taking (=mother and infants were together at sleeping site before mother left the nest; Fig. 1B) and Reunion (=mother returned to the infant at sleeping sites; Fig. 1B) to test the criteria suggested by Duranti^2^.

## Results

### Call rate of mothers and infants

**Figure 2:**
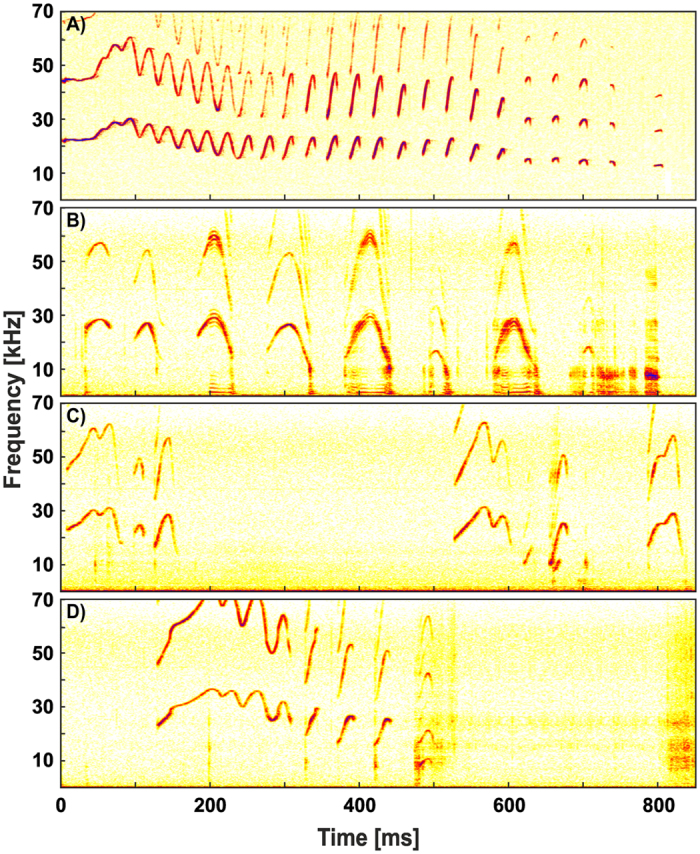
Spectrograms of mother and infant calls: (**A**) mother trill call (**B**) infant vocal streams with simple-modulated syllables, (**C**) infant vocal streams with multi-modulated syllables and (**D**) infant proto-trill.

**Figure 3:**
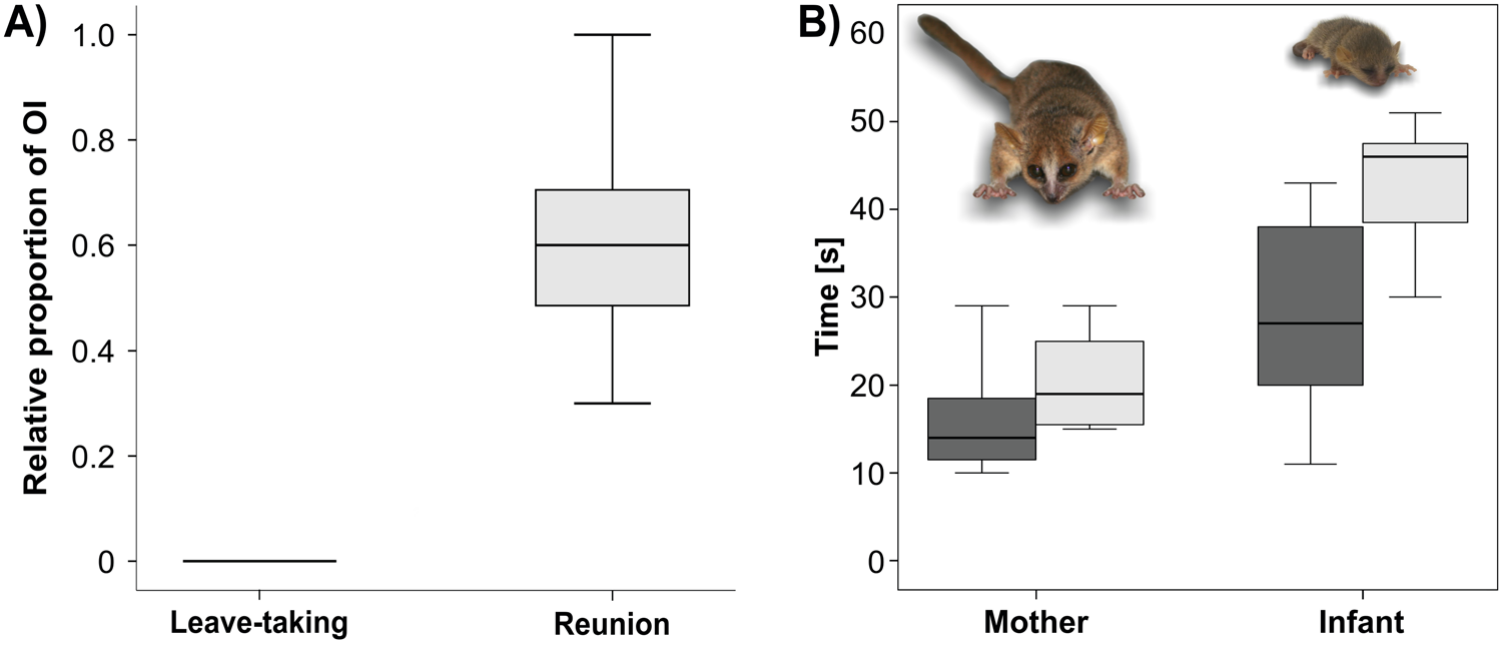
Greeting pattern in mother-infant interactions of mouse lemurs: (**A**) Comparison of reciprocal displays between the Leave-taking and Reunion condition, (**B**) Timing of mother and infant calls during the Reunion condition; line = median, box = 25–75% quartiles, whiskers = minimum-maximum, dark gray box = start time of first call, light gray box = start time of last call.

Mothers produced trill calls (Fig. 2A) whereas infants produced vocal streams (Fig. 2B–D) including vocalizations similar in acoustic shape to parts of the greeting call in the mother (proto-trill; Fig. 3D). All females (N = 11) produced trill calls in the Reunion condition, but four females produced trill calls in the Leave-taking condition. Thereby, general calling probability of mothers and infants was significantly higher in the Reunion compared to the Leave-taking condition (Mother: T = 0.00, N = 11, p = 0.003; Infants: T = 5.00, N = 11, p = 0.013; see Supplementary 1 – Table 1). This was also supported by a binomial GLMM analysis using subject and littersize as random factors (Mother: p < 0.001; Infants: p < 0.001).

### Reciprocal vocal exchange

Reciprocal sequences (percentage of OIs, where mother and infant were calling; see Supplementary 1 – Table 2) were significantly increased in the Reunion (median: 57.14%, interquartile range (=IR): 47.37–74.07%) compared to the Leave-taking condition (median: 0.00%; IR: 0.00–0.00%; T = 0.00, N = 11, p = 0.003; binomial GLMM: p < 0.001; Fig. 3A). The mother initiated 66.67% (=median; IR: 44.44–95.45%; see Supplementary 1 – Table 3) of these reciprocal exchanges which was not significant different from 50% chance level (T = 40, N = 11, p = 0.202). Sequential sequences (i.e. an alternate exchange of at least two infant and one mother calls or vice versa (e.g., mother-infant-mother or infant-mother-infant)) could be observed in 33.33% (=median, IR: 4.55–50.00) of the cases whereas antiphonal call sequences (i.e. an alternate exchange of at least two infant and two mother calls (e.g., mother-infant-mother-infant or infant-mother-infant-mother)) were rare (median: 11.11%, IR: 0.00–16.67%).

### Coordination of calling during Reunion in the nest

During Reunion, mothers started to vocalize significantly earlier than infants (T = 1, N = 11, p = 0.004; Fig. 3B; see Supplementary 1 – Table 3). The temporal distribution shows that mother trill calls occurred at the beginning of the reunion when the mother entered the sleeping box (median_start_: 13 s; median_end_: 19 s) whereas infants started calling after the mother had vocalized (median_start_: 25 s; median_end_: 47 s).

## Discussion

Mother-infant vocal exchanges in gray mouse lemurs fulfill all criteria of human greeting behavior established by Duranti^2^, therefore suggesting that patterns of human greeting behavior can be found in non-human mammals even with less complex social systems. Thus, observed vocal exchanges occur during Reunion but not during Leave-taking fulfilling the criteria of *context of encounter*. Thereby, vocal exchanges occurred at the start of the encounter fulfilling the criteria of *temporal unit of interaction*. During vocalizing the mother and the infant mouse lemurs had visual, olfactory and acoustic contact to each other fulfilling the criteria of *shared perceptual field*. The constant use of the same call types by the mother and infants fulfilled the criteria of *predictability*. Thereby mothers and infants used different call types (trill versus infant calls) which enabled *information about the interlocutors*. Mother and infants uttered vocalizations in the same time unit, showing that greeting displays fulfill the criteria of *reciprocal exchange*. Thus, our findings show that all criteria of human vocal greeting proposed by Duranti^2^ were fulfilled in mother-infant vocal exchanges in gray mouse lemurs.

Thereby, a striking difference between mouse lemurs, chimpanzees and macaques was the higher proportion of reciprocal exchanges in mouse lemurs (57% in comparison to 12% in chimpanzees^11^, 15.5% in Japanese macaques^22^). The reciprocal display is one criterion for human greeting behavior, thus unidirectional vocal displays of chimpanzees and macaques are not vocal greeting signals according to the criteria of Duranti^2^. Moreover, these vocal displays were uttered mainly by approaching higher-ranking group members^11, 20^. This suggests that in chimpanzees and macaques these signals might be more related to submissive displays to inhibit aggression. In contrast, howler monkeys use vocal reciprocal exchanges also in affiliative contexts^21^. Thereby, these vocal exchanges were the most common affiliative interaction between males and suggest that they have an important function in strengthening the social bonds among males^32^. The importance of social affiliation was also shown for chimpanzees, where the occurrence of reciprocal vocal exchanges depends on the strength of the social bond between both interaction partners^11^. Thus, for chimpanzees which had a strong affiliation to each other the probability for reciprocal vocal exchanges increased. Therefore, it might be argued that unidirectional vocal displays may function to inhibit aggression whereas reciprocal vocal exchanges during greeting are important to strengthen the social bonds between two interaction partners.

The strongest social bond across mammals is the social bond between the parents and their offspring^33, 34^. Vocal communication thereby plays a crucial role since it is used for vocal recognition^35, 36^, to induce infant- or maternal behavior (e.g.^37, 38^), or to release hormones (e.g.^39, 40^). Mother and infant contact calls have been described in a variety of mammalian and colony-breeding bird species (e.g.^35, 36, 41–43^). Thereby, Farmer^44^ argued that parental care might be the key innovation which accounts for many convergent behavioral displays of birds and mammals such as the contact calls between parents and offspring. Thus, in species where parents and infant are temporarily separated, vocal displays to find, recognize and socially accept each other would be important for the survival of the infant. Indeed in our studied primate species such greeting patterns do occur between mother and infants, but they do also seem to persist into adulthood. Braune *et al*.^45^ showed that adult mouse lemur females utter trill calls during sleeping group formation but not during dispersal, thereby suggesting that the trill call persists as a greeting signal also outside the mother-infant context. In our study, infants responded to mother calls by producing vocal streams with high vocal plasticity, including vocalizations similar in acoustic shape to parts of the greeting call in the mother (proto-trill; Fig. 3D). This suggests that the adult trill call develops during infancy and that parental interactions may be important for infant vocal development as shown in marmosets^46–50^ and gibbons^51^. Thus, during parent-infant reunions parental calls might be used as auditory templates to develop adult structure but also to ritualize the vocal communicative “greeting” function as suggested for human infants^52, 53^.

Our findings in a basal solitarily living primate show that the pre-human origin of the vocal greeting is evolutionarily deeply rooted in mammalian evolution and is independent from the degree of species sociality. In human greeting rituals vocal and gestural greeting displays can be used independently from each other (e.g., vocal: telephone opening^8^, gestural: waving from a distance^7^, facial: smiling^3^), but are generally combined^2–5^. Thereby, it is argued that the multimodal use is advantageous for transmitting more complex information which is especially important for species living in a complex and cohesive social system (*Multiple Message hypothesis* ^54, 55^). Thus, especially in group-living primates multimodal greeting displays evolved to predict more efficiently the behavior and intention of group-members (e.g., friendly approach, aggressive approach). Indeed complex systems of gestural communication also involved in greeting behavior are mainly observed in group- or pair-living monkeys and apes^56^. In contrast, mouse lemurs, like most non-primate mammals, are constrained in displaying complex gestural and facial displays^54^ limiting the use of the visual and tactile modality as greeting signal in comparison to monkeys and apes. Olfactory communication may also play an important role in recognition, but chemical cues are long-lasting^57^ and thereby not flexible enough to be used as a greeting signal. Thus, the evolutionary origin of human greeting behavior may be located in the unimodal use of vocal exchanges whereas multimodal greeting displays integrating gestural displays and facial expressions^58^ evolved later in primate evolution (when social systems became more complex). Thus, group-living combined with a longer life history (longer gestation period, a longer dependency from their parents and a longer life span^31^) in monkeys and apes compared to solitary ranging basal primates with a rapid life history may favor the development of complex multimodal greeting signals to challenge the social complexity of group-living. Thereby, sequentially organized behavioral patterns during parent-infant interaction will become ontogenetically ritualized to coordinate the social interactions of adult conspecifics. Thus, for both vocal and gestural displays parent-infant interactions play an essential role to acquire these communicative exchanges which are proposed to follow similar patterns of human conservation^19, 46–49, 59, 60^.

## Methods

### Subjects and data recording

We recorded the vocalizations of eleven family groups of gray mouse lemurs (*Microcebus murinus;* females with their offspring) at their sleeping sites for 24 hours at day 10 and 11 after birth. One female had one infant, seven females had twins and three females had triplets. Animals were housed in the breeding colony at the Institute of Zoology, University of Veterinary Medicine Hannover (housing details see^30, 61^). Females and infants were kept and observed in their home cages made of wire mesh (mesh was 1 × 1 cm; width × depth × height: 0.5 × 0.8 × 1.5 m; Bioscape GmbH, Castrop-Rauxel, Germany) including at least one sleeping box (20 × 12 × 15 cm). Each sleeping box was equipped with an ultrasonic microphone (SMX-US Weatherproof; Wildlife Acoustic, Inc., Concord, MA, USA), an infrared-emitting LED fixed to the ceiling and an infrared video camera (Sony Model 2005 XA B/W Ex view, Sony Corporation, Tokyo, Japan) filming through a clear plastic wall at the long side of the box. The microphones of two neighboring sleeping boxes were linked to a Song Meter SM2 Bat + recorder (Wildlife Acoustic, sampling frequency: 192 kHz per channel) resulting in a stereo-wave file. Videos were recorded with 25 frames per second using 4CH Full D1 DVR Network recording devices (RF Concepts Ltd., Dundonald, United Kingdom). An external acoustic signal that was recorded by the microphones of both the Song Meter and the infrared video camera was used to synchronize the audio files with the respective video files.

### Video and Audio analysis

To define the observation intervals for the Reunion and Leave-taking condition (Fig. 1A), we first analyzed the video material using The Player Lite HJ (AVTECH PlayerLiteHJ, AVTECH) for two behavioral events: (1) the time at which the mother enters the nest box (=mother is with all four limbs in the nest box) and (2) the time at which the mother leaves the nest box (mother is with all four limbs outside the nest box). In a pre-analysis, we noticed that mothers sit in front of the nest and call inside before entering the nest with all four limbs. To consider this approaching phase of the mother, the observation intervals (OIs) of the Reunion condition started the last 10 seconds before the mother entered the nest box and finished 50 seconds after entering. Further, to be sure that the absence of the mother was long enough to induce reunion behavior, mothers had to be at least 3 minutes absent from the nest box. The Leave-taking condition was defined as the 60 seconds before a mother leaves the nest. To be sure that mothers and infants stayed long enough together to observe mother-infant interactions, the mother had to be in the nest box for at least 2 minutes. For both conditions observation intervals (OIs) of one minute were defined and at least one infant had to be inside the nest box. In total, we analyzed 219 OIs for the Reunion condition and 429 OIs for the Leave-taking condition which met the aforementioned criteria.

To quantify calling behavior within the nest box, we inspected the spectrograms (Window size: 512; window type: Hanning) of the audio files for each OI visually using the audio-editor Audacity (2.0.1, 2.0.3. freeware, http://audacity.sourceforge.net). Vocalizations in the nest box were identified based on descriptions of the gray mouse lemur adult and infant vocal repertoire by Zimmermann^23^ and Scheumann *et al*.^27^. For each OI it was counted whether a mother call and/or an infant call occurred. Additionally, the time points of these calls were noted.

### Statistical analysis

To compare the calling probabilities between the Reunion and the Leave-taking condition, we calculated for each family group and each condition the percentage of OIs which contained at least one (1) mother call, (2) infant call and (3) both, mother and infant calls ( = reciprocal calling; see Supplementary 1 – Table 1–3). Since data were not normally distributed, we used the non-parametric Wilcoxon test for pairwise comparison and calculated the median and the interquartile range (25–75% quartile). To account for differences in littersize as well as to a different number of OIs per family, we conducted additionally a binomial GLMM using family and littersize as random factors (R version 3.1.1 (2014–07–10); R Core Team, 2014 “lme4” package; see Supplementary 1 – Table 4).

To investigate the initiator of the reciprocal vocal interactions in the Reunion condition, we calculated for each family group the percentage of OIs where the mother vocalized first followed by the infant. To test whether the mother initiated significant more interactions than expected by chance, we performed an one sample Wilcoxon test). Additionally, we calculated the percentage of OIs showing sequential or antiphonal calling. Sequential calling was defined as an alternate exchange of at least two infant and one mother calls or vice versa (e.g., mother-infant-mother or infant-mother-infant). Antiphonal calling was defined as an alternate exchange of at least two infant and two mother calls (e.g., mother-infant-mother-infant or infant-mother-infant-mother).

To analyze the timing pattern, we calculated for each family group the average time point of the first and last mother call and the first and last infant call, including all reciprocal OIs (see Supplementary 1 – Table 5).

All statistics were performed using SPSS 24 or R. The level of significance was set to p ≤ 0.05.

### Ethical approval and informed consent

The animal husbandry fulfilled all recommendations and was approved by the local veterinary authority (Lower Saxony State Office for Consumer Protection, Food Safety, and Animal Welfare Service, 42500/1 H). All observations were performed in accordance with the animal care guidelines of the European Directive 2010/63/EU and the applicable national laws in Germany. Since observations were performed in the housing cages no specific permission was required.

### Data availability

Audio and video files are stored in the data archive of the Institute of Zoology, University of Veterinary Medicine Hannover. The datasets generated during and/or analyzed during the current study are available from the corresponding author on reasonable request. A supplementary file includes all raw data used in this manuscript.

## Electronic supplementary material

Supplementary information 
